# Pharmacokinetics/pharmacodynamics of polymyxin B in patients with bloodstream infection caused by carbapenem-resistant *Klebsiella pneumoniae*

**DOI:** 10.3389/fphar.2022.975066

**Published:** 2022-12-16

**Authors:** Zhenwei Yu, Xiaofen Liu, Xiaoxing Du, Huiying Chen, Feng Zhao, Zhihui Zhou, Yu Wang, Yang Zheng, Phillip J. Bergen, Xi Li, Renhua Sun, Li Fang, Wanzhen Li, Yaxin Fan, Hailan Wu, Beining Guo, Jian Li, Yunsong Yu, Jing Zhang

**Affiliations:** ^1^ Sir Run Run Shaw Hospital, School of Medicine, Zhejiang University, Hangzhou, China; ^2^ Institute of Antibiotics, Huashan Hospital, Fudan University, Shanghai, China; ^3^ Zhejiang Provincial People’s Hospital, Hangzhou, China; ^4^ Biomedicine Discovery Institute and Department of Microbiology, Monash University, Melbourne, VIC, Australia; ^5^ Phase I Clinical Trial Center, Huashan Hospital, Fudan University, Shanghai, China

**Keywords:** polymyxins, pharmacokinetics/pharmacodynamics, CRKP, bloodstream infection, neurotoxicity, nephrotoxicity, breakpoint

## Abstract

**Introduction:** Polymyxin B is a last-line therapy for carbapenem-resistant microorganisms. However, a lack of clinical pharmacokinetic/pharmacodynamic (PK/PD) data has substantially hindered dose optimization and breakpoint setting.

**Methods:** A prospective, multi-center clinical trial was undertaken with polymyxin B [2.5 mg/kg loading dose (3-h infusion), 1.25 mg/kg/12 h maintenance dose (2-h infusion)] for treatment of carbapenem-resistant *K. pneumoniae* (CRKP) bloodstream infections (BSI). Safety, clinical and microbiological efficacy were evaluated. A validated liquid chromatography-tandem mass spectrometry (LC-MS/MS) method was applied to determine the concentrations of polymyxin B in blood samples. Population pharmacokinetic (PK) modeling and Monte Carlo simulations were conducted to examine the susceptibility breakpoint for polymyxin B against BSI caused by CRKP.

**Results:** Nine patients were enrolled and evaluated for safety. Neurotoxicity (5/9), nephrotoxicity (5/9), and hyperpigmentation (1/9) were recorded. Blood cultures were negative within 3 days of commencing therapy in all 8 patients evaluated for microbiological efficacy, and clinical cure or improvement occurred in 6 of 8 patients. C_max_ and C_min_ following the loading dose were 5.53 ± 1.80 and 1.62 ± 0.41 mg/L, respectively. With maintenance dosing, AUC_ss,24 h_ was 79.6 ± 25.0 mg h/L and C_ss,avg_ 3.35 ± 1.06 mg/L. Monte Carlo simulations indicated that a 1 mg/kg/12-hourly maintenance dose could achieve >90% probability of target attainment (PTA) for isolates with minimum inhibitory concentration (MIC) ≤1 mg/L. PTA dropped substantially for MICs ≥2 mg/L, even with a maximally recommended daily dose of 1.5 mg/kg/12-hourly.

**Conclusion:** This is the first clinical PK/PD study evaluating polymyxin B for BSI. These results will assist to optimize polymyxin B therapy and establish its breakpoints for CRKP BSI.

## Introduction

The increasing global prevalence of carbapenem-resistant Gram-negative organisms (CRO) represents a major threat to health (WHO, 2021). In China alone, ∼20%–30% of *Pseudomonas aeruginosa* and *Klebsiella* spp. and >70% of *Acinetobacter* spp. are carbapenem resistant (http://www.chinets.com/), and such infections are associated with high morbidity and mortality (Gu et al., 2018; Wang et al., 2018). Carbapenem-resistant *K. pneumoniae* (CRKP) is a particular problem, with pooled mortality among patients infected with CRKP reported to be approximately 42% and mortality among bloodstream infection (BSI) patients estimated to exceed 50% (Xu et al., 2017). Although new drug combinations such as ceftazidime-avibactam have shown good clinical efficacy against KPC-producing *K. pneumoniae*, treatment options remain limited (Wright et al., 2017). Polymyxin B may be the only accessible or affordable therapeutic option for CRKP in many countries (Satlin et al., 2020).

While use of polymyxin B for the treatment of CRKP is increasing, a lack of pharmacokinetic/pharmacodynamic (PK/PD) evidence in support of appropriate susceptibility breakpoints has hindered its clinical use (Medeiros et al., 2019). Susceptibility breakpoints for polymyxin B are difficult to determine from existing clinical studies given the large interpatient variability observed with polymyxin B exposures, varying sites of infection examined (e.g., lung and bloodstream), and a wide range of MIC values of the infecting pathogens (Tsuji et al., 2019). While organizations such as the European Committee on Antimicrobial Susceptibility Testing (EUCAST) provide a susceptible breakpoint category for colistin (polymyxin B breakpoints are not reported) (Version 12.0, 2022), in 2020 the Clinical and Laboratory Standards Institute (CLSI) removed the susceptible interpretive category for the polymyxins (previously ≤2 mg/L in all cases) (Satlin et al., 2020). The decision by the CLSI is not rational as it was primarily based on the data that showed polymyxins are not efficacious for the treatment of lung infections in mice and patients following intravenous administration (Rigatto et al., 2013; Cheah et al., 2015; Landersdorfer et al., 2018; Satlin et al., 2020; Nang et al., 2021). Both EUCAST and CLSI agree that polymyxins should have clinical breakpoints for Enterobacterales, *P. aeruginosa* and *Acinetobacter baumannii*; and have highlighted the urgent need for clinical PK/PD data to support the establishment of breakpoints and optimize the dosage regiments of polymyxins (Satlin et al., 2020).

Ideally, susceptibility breakpoints of antibiotics should be determined based on their PK/PD at the infection site and administration route (Bian et al., 2022); however, clincial investigations on the efficacy of intravenous polymyxins in the literature are from mixed types of infections predominantly with pneumonia (Paul et al., 2018; Almangour et al., 2021). Notably, favorable PK/PD relationships of colistin and polymyxin B have been reported in neutropenic mouse thigh infection models (Cheah et al., 2015; Landersdorfer et al., 2018). Here, we investigated the clinical PK/PD of intravenous polymyxin B (2.5 mg/kg loading dose and maintenance dose of 1.25 mg/kg 12-hourly) in patients with CRKP bloodstream infection using an intensive sampling strategy. Both microbiological and clinical efficacy were evaluated to determine the dose-response relationship of polymyxin B. Our study aims to provide clinical evidence for the rational determination of PK/PD driven susceptibility breakpoints of polymyxins in patients with bloodstream infection.

## Materials and methods

### Study design and ethics approval

This prospective, multi-centered, single-armed and open-label clinical trial was conducted in accordance with Good Clinical Practice, International Conference on Harmonization guidelines, and in compliance with the World Medical Association Declaration of Helsinki and was approved by the Ethics Committee of Sir Run Run Shaw Hospital, College of Medicine, Zhejiang University, Hangzhou, China, and other participating centers (Williams, 2008). Written informed consent was obtained from each patient or his/her representative before enrollment. Patients with CRKP-positive blood cultures and with an estimated glomerular filtration rate (eGFR) of 60–120 ml/min were included. The inclusion and exclusion criteria are shown in Supplementary Table S1. This clinical trial was registered at http://www.chictr.org.cn (registration number: ChiCTR 1900021137) and was conducted across 2019 and 2020.

### Drug administration

Polymyxin B Sulphate for Injection (Lot No. A-1411137) containing 72.0% polymyxin B1 and B1-Ile and 12.1% polymyxin B2 was provided by SPH No. 1 Biochemical & Pharmaceutical Co., Ltd. (Shanghai, China). Prior to administration, polymyxin B was dissolved in 50–100 ml normal saline as per the manufacturer’s instructions then administered intravenously at a dose of 1.25 mg/kg every 12 h (2-h infusion) with an initial loading dose of 2.5 mg/kg (3-h infusion). Treatment lasted for 7–14 days as per treatment guidelines (Rhodes et al., 2017). Adverse events were closely monitored and recorded for each patient.

### Susceptibility testing

The minimum inhibitory concentrations (MICs) to polymyxin B and meropenem of the collected CRKP strains were determined by broth microdilution according to the European Committee on Antimicrobial Susceptibility Testing (EUCAST) guidelines and interpreted according to EUCAST breakpoints of colistin (Breakpoint tables for interpretation of MICs and zone diameters, Version 11.0); *Escherichia coli* ATCC 25922 was used as the control strain. Polymerase chain reaction (PCR) was employed to determine the presence of resistance genes (KPC, NDM, IMP, OXA, VIM). The primers were the same as in a published protocol and the sample process procedures were adapted a little bit (Poirel et al., 2011). To be brief, a single colony of the bacteria were picked and resuspended in ultra-pure water, and total DNA was extracted by heating bacterial suspension at 100°C for 10 min to lysis and release DNA. The suspension was centrifuged for total DNA which was dissolved in supernatant. Then 2 µl total DNA, 1 µl forward and 1 µl reverse primers (10 µM) for each targeted gene, 12.5 µl 2 × Hieff^®^ Robust PCR Master Mix (Yeasen, Shanghai, China) and ultra-pure water were mixed into a 25 µl reaction mixture. The mixture was subjected to amplification process, which was 5 min at 94°C, and 30 cycles of amplification consisting of 30 s at 94°C, 30 s at 55°C, and 50 s at 72°C, with 5 min at 72°C for the final extension. Finally amplified DNA fragments were analyzed by electrophoresis in a 1% agarose gel. Multilocus sequence typing (MLST) analysis of the isolates was performed following Seemann T, mlst (https://github.com/tseemann/mlst), according to PubMLST website (Jolley et al., 2018).

### Safety and clinical efficacy

The definition of different population groups for safety and efficacy analysis is shown in Supplementary Table S2. Briefly, safety was evaluated based on adverse events (AEs) in patients who received at least one polymyxin B dose. Adverse events were recorded based on patient self-reporting as well as abnormal laboratory test results including serum chemistry and routine blood and urinary testing. Acute kidney injury (AKI) was graded according to the RIFLE criteria (Bellomo et al., 2004). The primary endpoint was culture-confirmed bacterial eradication of CRKP from the blood evaluated by blood cultures taken every 3 days following the first dose of polymyxin B administration. Secondary endpoints were clinical outcome (clinical cure or improvement, Supplementary Table S3) evaluated in patients who received at least 3 days of polymyxin B treatment, and 28-day all-cause mortality evaluated in patients who completed 14 days of polymyxin B treatment.

### Determination of polymyxin B concentrations in blood samples

Blood samples (4 mL) for pharmacokinetic analysis were collected in tubes with EDTA-K2 as the anticoagulant immediately before and after administration of the polymyxin B loading dose (the first dose) and immediately before administration of the second dose. Subsequent blood samples were collected during the second visit (5th, 6th or 7th dosing) consisting of a sample immediately prior to the infusion and at .5, 1, 2, 3, 4, 5, 6, 7, 8, 10, and 12 h following the commencement of the infusion. If possible, a final blood sample was collected 24 h after the commencement of the last infusion. All blood samples were centrifuged (4°C, 2,000 *g*) and plasma collected and stored at −80°C until analysis.

The concentrations of polymyxin B1 (including B1-Ile) and polymyxin B2 were determined using a validated liquid chromatography-tandem mass spectrometry (LC-MS/MS) method (Liu et al., 2020). Calibration and quality control (QC) samples were prepared using USP standard polymyxin B. This method showed excellent linearity (average *R* ^2^ = 0.9931), precision (3.2%–10.0%), and accuracy (91.1%–105.1%). The calibration range was .050–5.00 mg/L for polymyxin B1 and .011–.549 mg/L for polymyxin B2. The concentration of polymyxin B was calculated by the sum of concentrations of polymyxin B1 and B2 using molar terms and their molecular weights (polymyxin B1 is 1203.48 g/mol, polymyxin B2 is 1189.45 g/mol).

### PK analyses and population pharmacokinetic (PPK) modeling

The PK parameters of polymyxin B were calculated using Phoenix WinNonlin 8.0 (Certara^TM^, USA) with non-compartmental and compartmental methods. PPK analysis was conducted using NONMEM 7.4 (Icon Development Solutions, Ellicott City, MD) with G77 FORTRAN complier and FOCEI algorithm. Results from BSI patients in the present study and healthy subjects in a previous study were combined (Liu et al., 2021). One- and two-compartment models were tested during development of the base model. Models were developed and evaluated based on an objective function value and goodness-of-fit plots. The covariates were selected using a forward inclusion and backward elimination strategy. The screened covariates were sex, age, body weight, height, body mass index (BMI), creatinine clearance (CLcr), albumin, hemoglobin, total protein, total bilirubin, serum alanine aminotransferase (ALT), serum aspartate aminotransferase (AST), serum creatinine, Sequential Organ Failure Assessment (SOFA) score, and disease state (BSI patients were defined as 1 and healthy subjects were defined as 2). Covariates were included in the model based on the criteria of OFV requiring a decrease of 3.84 (*p* < .05) in the forward inclusion and an increase of greater of 10.83 (*p* < .001) in the backward elimination. Bootstrap sampling and visual predictive checks (VPCs) were utilized to validate the robustness of the final model.

### PK/PD analysis and simulations

Monte Carlo simulations were conducted for the probability of target attainment (PTA) analysis to guide the selection of optimal dosage regimens. Different dosage regimens were simulated by the PPK model and AUC_ss,24h_ were calculated. An unbound fraction (*f*) of polymyxin B in human plasma of 42% (Sandri et al., 2013) was used to calculate the *f*AUC_ss,24h_/MIC (the PK/PD index that best correlates with optimal microbiological outcomes) at steady state (Dudhani et al., 2010b; 2010a; Bergen et al., 2010; Cheah et al., 2015; Landersdorfer et al., 2018). The maximal reductions in colony forming units (CFU)/thigh derived from murine thigh infection models using polymyxin B or colistin were set as PK/PD targets. Specifically, *f*AUC_24h_/MIC targets of 17.4 for polymyxin B for a 1-log_10_ reduction against *K. pneumoniae* (Landersdorfer et al., 2018), and 13.5 and 17.6 for colistin for a 2-log_10_ reduction against *P. aeruginosa* and *A. baumannii*, respectively, were used in the Monte Carlo simulations (Cheah et al., 2015). The colistin targets were adopted for polymyxin B given that both polymyxins have essentially identical *in vitro* potencies (as measured by MICs), spectra of antibacterial activity and efficacy against thigh infection in mice (Landersdorfer et al., 2018; Satlin et al., 2020).

## Results

### Patient enrollment and adverse events

Nine Chinese patients (seven males and two females) were enrolled in the present study, with four patients receiving the full 14 days of polymyxin B treatment (Table 1). Baseline demographic data including SOFA score and original infection sites are shown in Table 2, and the co-administered drugs are shown in Supplementary Table S4. All nine patients were administered at least one dose of polymyxin B and all were included in the safety analysis. Adverse events are summarized in Table 3. At least one of neurotoxicity (5/9), nephrotoxicity (5/9), and hyperpigmentation (1/9) were recorded in 88.9% (8/9) of patients. Five of seven conscious patients (two patients being sedated during the treatment period) reported neurotoxicity. Of these patients, four reported pruritus and paresthesia on the face and head after the polymyxin B loading dose while three were evaluated as ataxic with the symptoms resolving by days 3–5 without any additional treatment. Five patients (four males and one female) experienced an increase in serum creatinine within 7 days, with all graded “I” according to the RIFLE criteria. Of these five patients, one (patient 7) continued in the study and completed 14 days of treatment, three (patients 1, 2 and 8) withdrew from the study and continued on a reduced polymyxin B dose (50 mg, 12-hourly), and one (patient 5) withdrew from the study and stopped polymyxin B treatment on day 6. The changing trends in eGFR and serum creatinine for all patients across the study period are shown in Supplementary Table S5. Hyperpigmentation on the head and neck was observed in patient 9 on day 4 which did not resolve until after the study was completed on day 28. Unlike in healthy subjects, no abdominal pain was reported by any patient during polymyxin B treatment (Liu et al., 2021).

**TABLE 1:** Polymyxin B MICs, pharmacokinetic/pharmacodynamic indices, and clinical outcome.

Patient no.	*K. pneumoniae* strain no.	Polymyxin B MIC (mg/L)	Treatment duration (day)	Study completion per protocol (Withdrawal reasons)	Continued polymyxin B treatment after withdrawal	AUC24 h,ss/MIC	Microbiological efficacy	Clinical efficacy^**^	28-day survival
1	1	.5	6	Withdraw (AKI)	Yes	110.4	Eradication	Not improved	-
2	2–1, 2–2*	.5, .5	6	Withdraw (AKI)	Yes	266.8	Eradication	Improved	-
3	3	1	14	Yes	-	68.8	Eradication	Cure	Yes
				Withdraw (pruritus and numbness on face and head, ataxia)					
4#	4	.5	1		No	-	-	-	-
				Withdraw (AKI, pruritus and numbness on face and head, ataxia)					
5	5	.5	5		No	172	Eradication	Improved	-
6	6	.5	14	Yes	-	149.6	Eradication	Cure	Yes
7	7	1	14	Yes	-	87.8	Eradication	Cure	Yes
				Withdraw (AKI, pruritus and numbness on face and head, ataxia)					
8	8	.5	4		Yes	154.4	Eradication	Improved	-
9	9	1	14	Yes	-	54.4	Eradication	Failure	Yes

MIC, minimum inhibitory concentration; AUC24 h,ss, area under the plasma concentration-time curve across 24 h at steady state; *, two CRKP strains were isolated; -, not applicable or not evaluated; **, evaluated when the subjects withdrew or completed the study; ^#^, the PK/PD and efficacy could not be evaluated as the patient withdrew after receiving only the loading dose. All strains had a meropenem MIC of >64 mg/L except for strain No. 2–2 (MIC of 4 mg/L).

**TABLE 2:** Baseline characteristics of enrolled patients.

Characteristics	*n* (%) or median (IQR) (*N* = 9)
Age (year)	68 (63–73)
Sex	
Male	7 (78)
Female	2 (22)
Weight (kg)	60 (55–65)
Body mass index (kg/m^2^)	22.2 (17.6–24.4)
Serum creatinine (μmol/L)	61 (52–73)
Creatinine clearance (ml/min)	89 (68–106)
Serum albumin concentration (g/L)	28.8 (26.9–30.3)
SOFA score	4 (2–5)
Original infection site	
Intra-abdominal infection	6 (67)
Testicular infection	1 (11)
Urinary tract infection	1 (11)
Cather related blood infection	1 (11)

IQR, interquartile range; SOFA, sequential organ failure assessment.

**TABLE 3:** Adverse events following intravenous administration of polymyxin B in patients with CRKP bloodstream infections.

Adverse effect	Event
Total	16
Neurotoxicity	
Pruritus	4
Ataxia	2
Dizziness	2
Weakness	1
Numbness of extremities	1
Nephrotoxicity	
Acute kidney injury (Grade I)	5
Other	
Skin hyperpigmentation	1
Back pain	1

### Pharmacokinetics of polymyxin B in BSI patients

**FIGURE 1:**
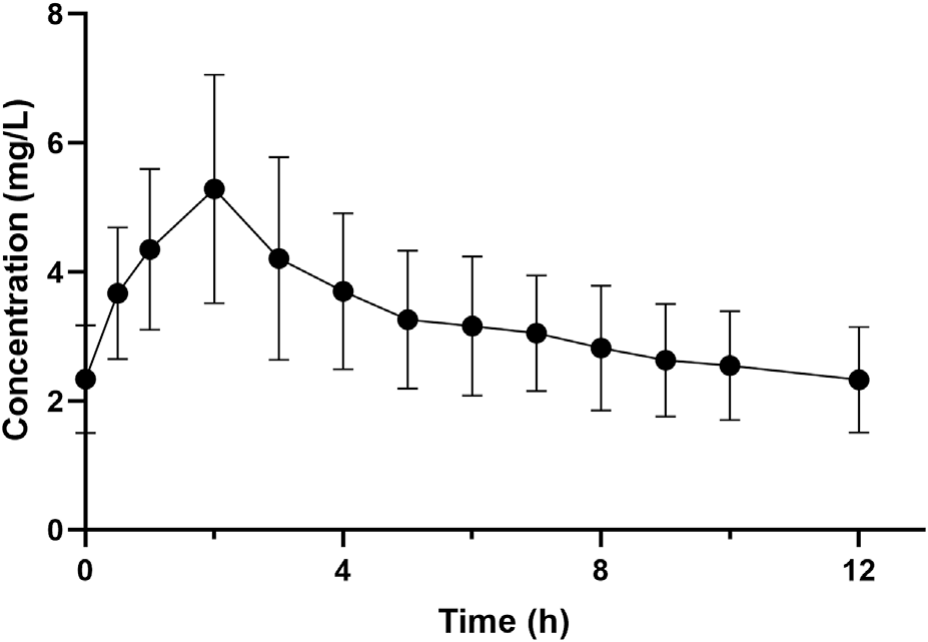
Mean (±SD) plasma concentration-time profile of polymyxin B at steady state in patients with carbapenem-resistant *K. pneumoniae* bloodstream infections. The *X*-axis is normalized to the dosing time at steady state. Time “0 h” is equivalent to immediately before polymyxin B dosing with the remaining times calculated from the commencement of dosing at steady state.

The average concentration–time profile of polymyxin B at steady state across all patients is shown in Figure 1. The mean (±SD) C_max_ and C_min_ (12 h) following the loading dose was 5.53 ± 1.80 mg/L and 1.62 ± 0.41 mg/L, respectively. The corresponding PK parameters of polymyxin B derived from non-compartmental analyses at steady state are shown in Table 4. The pharmacokinetics of polymyxin B was best described by a two-compartmental model with the parameters shown in Supplementary Table S6.

**TABLE 4:** Pharmacokinetic parameters by non-compartmental analysis.

Parameter	Unit	Mean value ± SD
C_max_	mg/L	5.42 ± 1.69
AUC_12h_ ^*^	mg·h/L	39.8 ± 12.5
C_avg_	mg/L	3.35 ± 1.06
T_1/2_	h	12.5 ± 3.11
CL	L/kg/h	0.028 ± 0.007
V_d_	L/kg	0.490 ± 0.142

SD, standard deviation; AUC_12h_, area under the concentration-time curve across 12 h; Cavg, average concentration; T1/2, half-life; CL, clearance; Vd, volume of distribution. All values are calculated at steady state.

* The corresponding AUC_24h_ (area under the concentration-time curve across 24 h) is 79.6±25.0 mg h/L.

### Microbiological and clinical efficacy

The polymyxin B MICs of ten *K. pneumoniae* strains isolated from the nine patients (two strains isolated from one patient) are shown in Table 1. All strains were susceptible to polymyxin B (MIC ≤1 mg/L) and were resistant to meropenem (MICs >64 mg/L for 9 strains and 4 mg/L for 1 strain). PCR results showed that all strains harbored *bla*-KPC2 and that New Delhi metallo-beta-lactamase (NDM), imipenem-resistant carbapenemase (IMP), Verona integron metallo-β-lactamase (VIM) or oxacillinase (OXA) genes were absent (Supplementary Figure S1). Except isolates from patient No. 8 belonged to ST14, all the rest isolates were belonged to ST11, which is the predominant clone type in ICU in China (Qin et al., 2020).

One patient withdrew from the study following the loading dose due to intolerable pruritus on the face and head and was excluded from the microbiological and clinical efficacy analysis. Blood cultures showed eradication of all isolated CRKP from the remaining 8 patients by the third day of treatment and remained negative until the patients completed the study or withdrew. Clinical cure or improvement occurred in 75% (6/8) of these patients, with all 4 patients who completed the 14-day polymyxin B treatment protocol alive 28 days after the commencement of therapy.

### Population PK and PK/PD analysis

**FIGURE 2:**
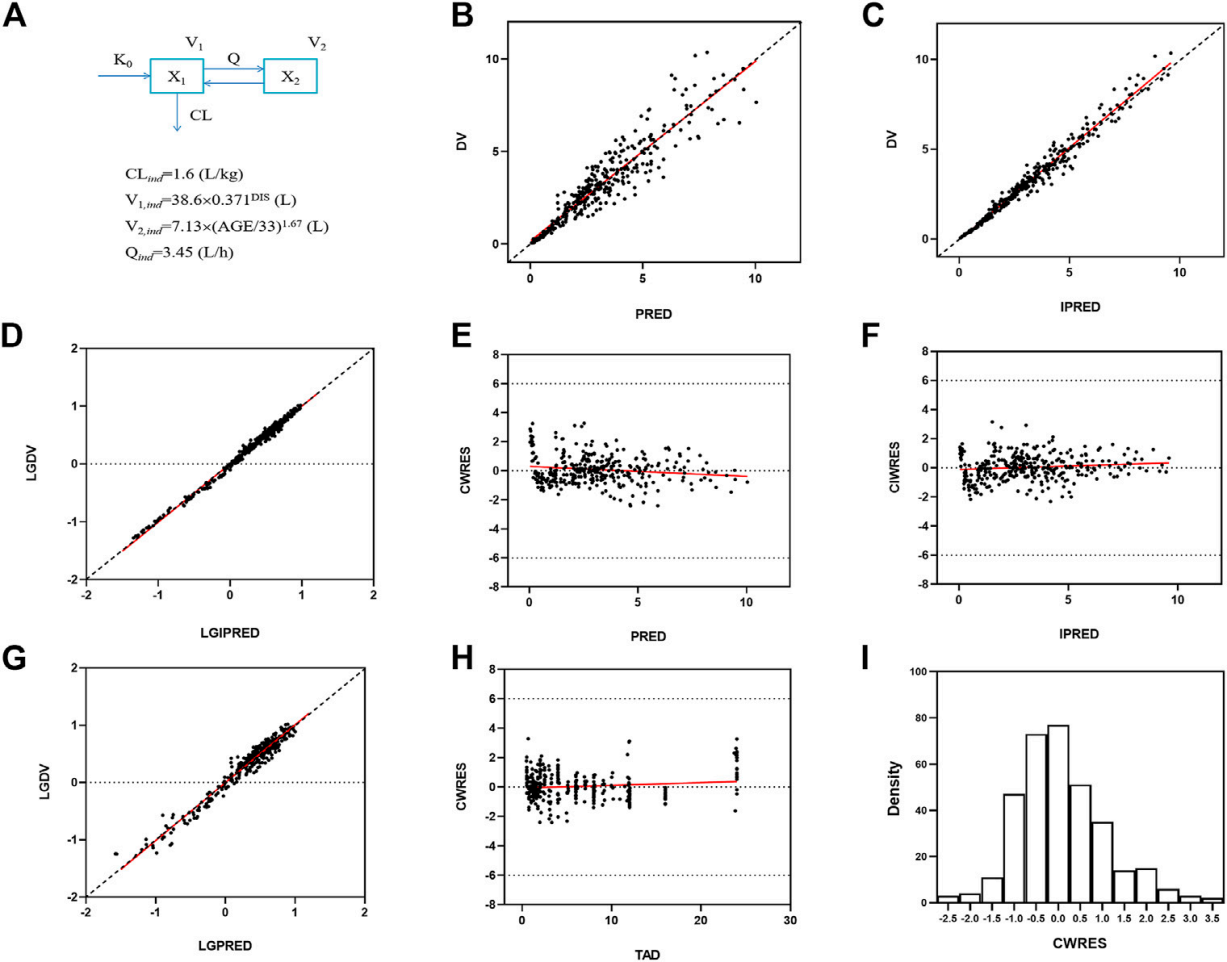
Goodness-of-fit plots for the final population PK model. Predicted concentrations are in micrograms per liter and time is in hours. **(A)** PPK model schematic figure and final model, DIS = 1 for patients and DIS = 2 for healthy subjects; **(B)** Observed polymyxin B concentrations (DV) *versus* population predictions (PRED). **(C)** DV *versus* individual predictions (IPRED). **(D)** Log transformed DV *versus* Log transformed IPRED. **(E)** Conditional weighted residuals (CWRES) *versus* PRED. **(F)** Conditional individual weighted residuals (CIWRES) *versus* IPRED. **(G)** Log transformed DV *versus* Log transformed PRED. **(H)** CWRES *versus* time after dose (TAD). **(I)** distribution of CWRES.

**FIGURE 3:**
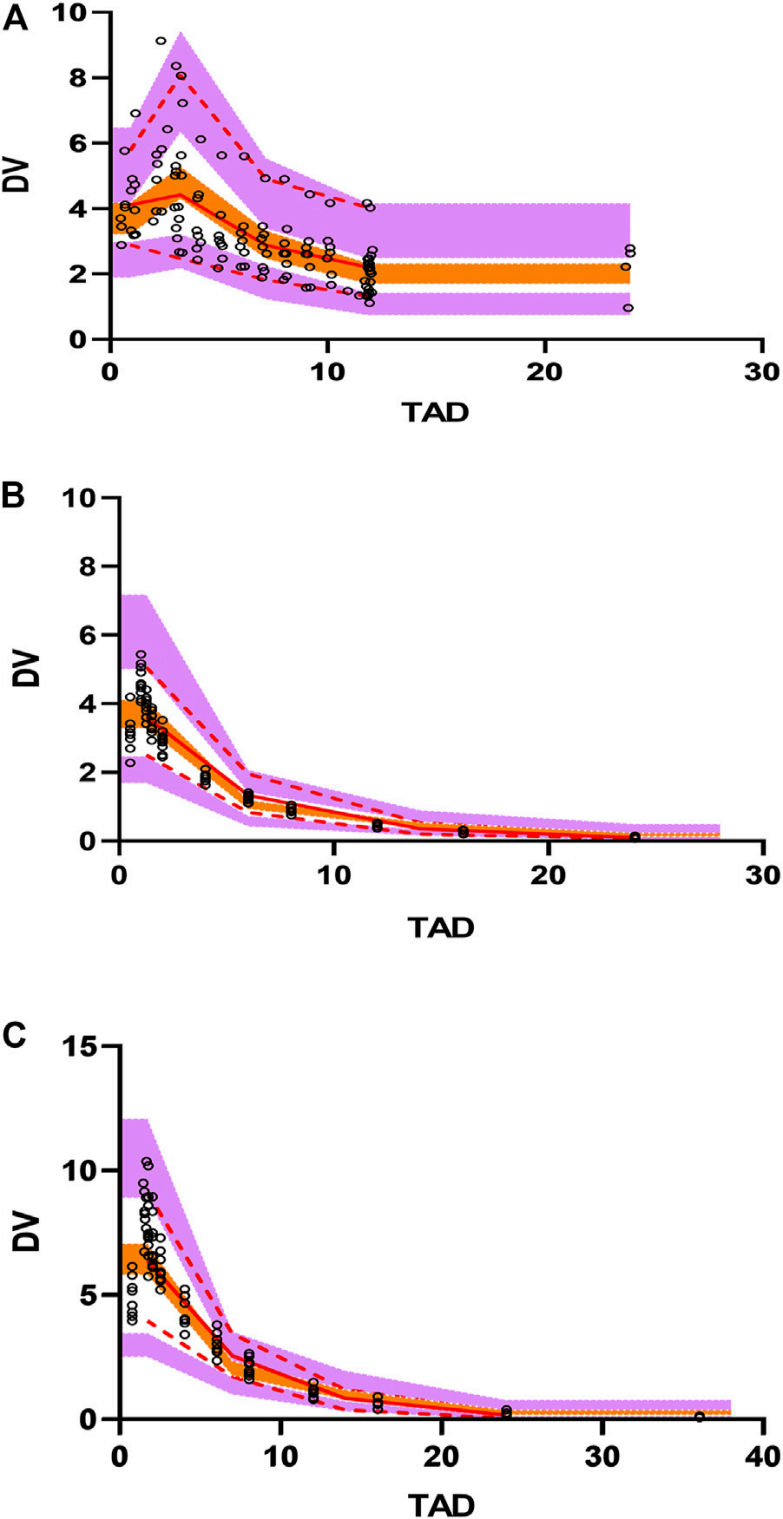
Visual predictive check (VPC) plots for the polymyxin B concentration in plasma. **(A)** VPC for BSI patients. **(B)** VPC for healthy subjects with .75 mg/kg dosing administration. **(C)** VPC for healthy subjects with 1.5 mg/kg dosing administration. Open circles represent observed concentrations. The solid red line represents the median of the observations. The red dashed lines represent the 5th and 95th percentiles of the observations. The purple shaded areas represent the 95% confidence intervals for the 5th and 95th percentiles, and the orange shaded areas represents the median of the predicted data.

The polymyxin B concentration data were fitted into a two-compartmental model with the OFV value of −988 during the base model development. An exponential model and a proportional model were used to describe the interindividual variability and residual variability, respectively. Two covariates [BSI on the volume of central compartment (V1) and age on the volume of peripheral compartment (V2)] were included in the final population PK model where the OFV value decreased from −988 to −1087. The final PPK model and parameters are shown in Figure 2, and the progression of model building was shown in Supplementary Table S7. The final model was evaluated by goodness-of-fit plots and showed no apparent visual bias for the predictions (Figure 2; Supplementary Figures S2, S3). The parameter estimates and between-subject variability from the final model and 1,000 bootstrap runs are presented in Table 5. Both bootstrap and VPC plots (Figure 3) indicated the robustness of the final model.

**TABLE 5:** Population pharmacokinetic parameter estimates and between-subject variability.

Parameter (Unit)	Estimate		Between-subject variability	
	Original dataset (Typical value)	Bootstrap dataset (Median and 95% interval confidence)	Original dataset (%)	Bootstrap dataset (%)
CL (L/h)	1.60	1.60 (1.5, 1.7)	18.2	17.7
V1 (L)	38.6	38.6 (27.7, 54.4)	20.0	18.7
V2 (L)	7.13	7.04 (5.93, 7.98)	27.2	25.6
Q (L/h)	3.45	3.39 (2.31, 4.48)	0 (FIX)	—
θDIS on V1	0.371	0.372 (0.31, 0.45)	NA	NA
θAGE on V2	1.67	1.68 (1.34, 1.95)	NA	NA
Proportional error (%)	11.8	11.6 (10.4, 12.9)	NA	NA

CL, clearance from the central compartment; V1, central volume of distribution; V2, peripheral volume of distribution; Q, intercompartmental clearance.

**FIGURE 4:**
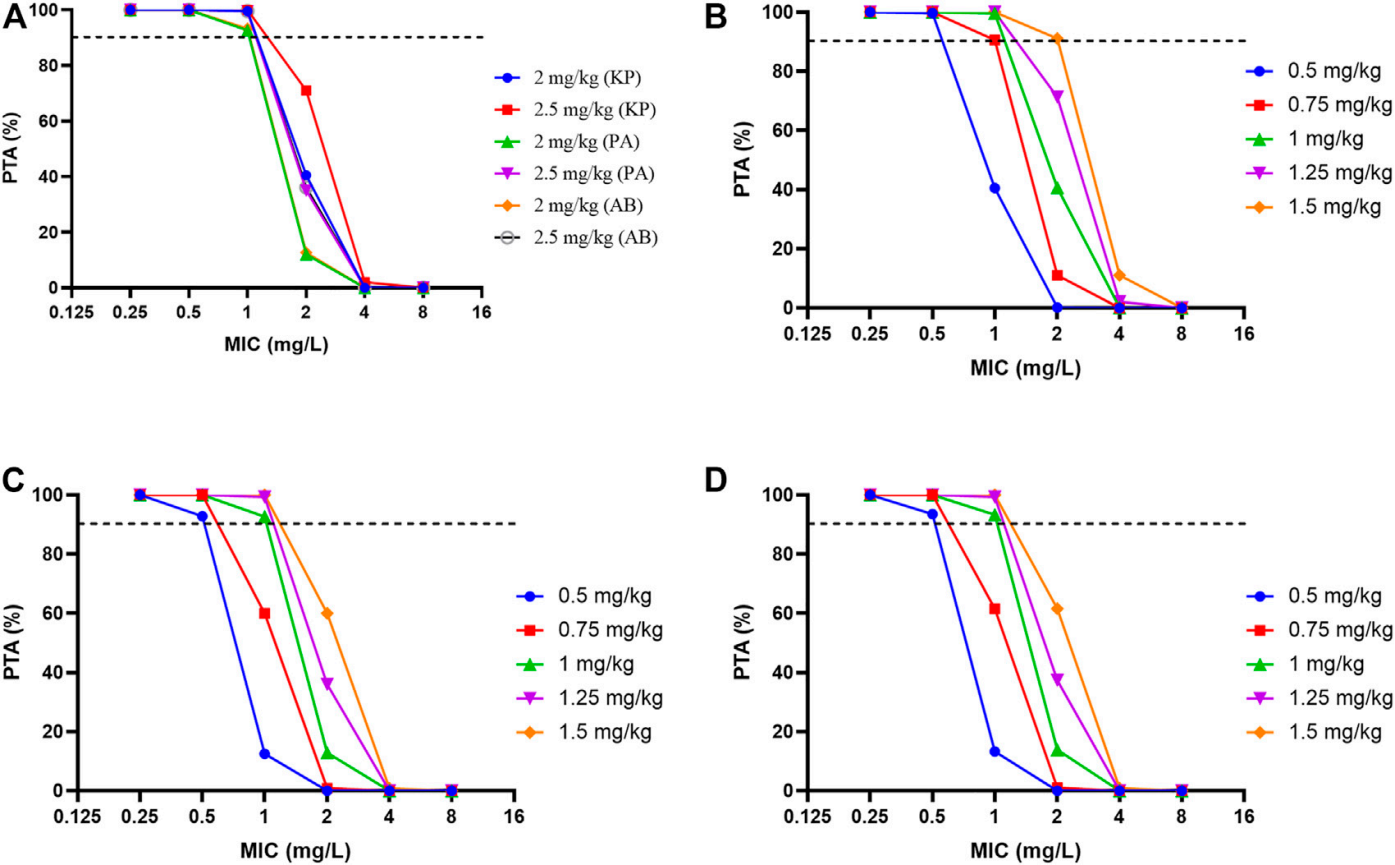
Probability of target attainment for different dosage regimens of polymyxin B against common carbapenem-resistant organisms according to PK/PD simulations. **(A)** Loading dose for three bacterial species. **(B)** *K. pneumoniae* (KP), **(C)** *P. aeruginosa* (PA), **(D)** *A. baumannii* (AB)*;* The *f*AUC_ss,24h_/MIC targets were 13.5 for a 1-log_10_ reduction against *K. pneumoniae* and 17.6 and 17.4 for a 2-log_10_ reduction against *P. aeruginosa* and *A. baumannii*, respectively. The unbound fraction in plasma was .42.

Probability of target attainment for different dosage regimens of polymyxin B against common carbapenem-resistant organisms according to PK/PD simulations was shown in Figure 4 and Supplementary Table S8. For *K. pneumoniae*, *P. aeruginosa* and *A. baumannii* with MICs ≤1 mg/L, Monte Carlo simulations of polymyxin B revealed >90% PTA for *f*AUC_ss,24h_/MIC targets with the 1 mg/kg dosing regimen (Figure 4). However, with an MIC of 2 mg/L the PTA with this regimen dropped below 50% for each organism and remained below 90% even with the maximum dose of 3 mg/kg/d (corresponding to the 1.5 mg/kg 12-hourly regimen).

## Discussion

In our prospective clinical PK/PD study, CRKP with a polymyxin B MIC ≤1 mg/L was eradicated from the bloodstream of Chinese patients with an achieved polymyxin B AUC_ss,24h_/MIC of ≥54.4 (equivalent to an *f*AUC_ss,24h_/MIC of ≥22.8). Neurotoxicity and nephrotoxicity were the major dose-limiting factors associated with polymyxin B administration. Intensive PK sampling and PPK modeling showed that disease status and age were covariates of V1 and V2, respectively. Monte Carlo simulations suggested that a polymyxin B dose of 1 mg/kg 12-hourly was effective for pathogens with an MIC ≤1 mg/L.

Nephrotoxicity following intravenous administration of polymyxin B may occur in up to ∼30% of patients and constitutes the major dose-limiting factor with polymyxin therapy (Ouderkirk et al., 2003; Sobieszczyk et al., 2004; Phe et al., 2014; Oliota et al., 2019). The inability to substantially increase the daily dose of polymyxins may lead to suboptimal dosing which compromises efficacy and increases the likelihood of resistance emerging (Zavascki et al., 2007; Rigatto et al., 2014). In the present study, no pathological factors affecting renal function were present in any of the participants prior to enrollment, and no concomitant nephrotoxic drugs were administered during the study period. Polymyxin B-induced AKI as defined by the RIFLE criteria occurred with an incidence of 55.6% (5/9) 4–7 days after commencing therapy; this incidence is approximately twice that reported previously (29.8%) (Oliota et al., 2019). This discrepancy might be at least partially explained by the different definitions of nephrotoxicity (e.g., RIFLE, AKIN, KDIGO) applied in earlier studies which also included a wide range of polymyxin B doses (12–225 mg/day) patient variability (baseline characteristics and clinical conditions), and variations in interventions being assessed. However, the study by Phe *et al.* used the same RIFLE criteria to define nephrotoxicity and reported a considerably lower incidence of polymyxin B-associated nephrotoxicity than we observed (23.1% vs. 55.6%) (Phe et al., 2014). The high dose of polymyxin B administered (2.5 mg/kg/day) in the present study could be a risk factor. Since polymyxin dose is known to be the most important variable associated with the development of nephrotoxicity, with total drug exposure and longer duration of treatment also associated with its development (Nation et al., 2019).

Neurotoxicity is another major dose-limiting factor for the polymyxins, although it often goes unreported given many patients receiving polymyxins are mechanically ventilated which makes assessment difficult. Polymyxin-induced neurotoxicity is very likely dose-dependent and is characterized by symptoms such as dizziness, vertigo, visual disturbances, confusion, hallucinations, seizures, ataxia, facial and peripheral parenthesis, and pruritus (Falagas and Kasiakou, 2006; Weinstein et al., 2009; Wahby et al., 2010; Honore et al., 2013). While neurologic side effects such as parenthesis have been reported to be as high as 27% with intravenous polymyxin therapy (Wallace et al., 2008), our recently published study examining polymyxin B toxicity in healthy Chinese patients reported perioral paraesthesia, dizziness, and numbness of extremities occurred in 7 of 10 (70%) subjects who received a single dose of 0.75 mg/kg *via* a 1-h infusion, and all subjects who received a single dose of 1.5 mg/kg *via* a 1.5-h infusion (Liu et al., 2021). The high incidence of neurotoxic adverse events (5 of 7 conscious patients) in the present study is thus similar to our earlier report. Unfortunately, it appears that there is considerable overlap in plasma concentrations required to achieve the desired antibacterial effect and those causing the major adverse effects of nephrotoxicity and neurotoxicity (Nation et al., 2019). While we had initially planned to include a higher-dose polymyxin B regimen (loading dose of 2.5 mg/kg with a maintenance dose of 3 mg/kg/d) in the research protocol for the present study, consideration of the potential for nephrotoxicity and neurotoxicity meant that the higher dose regimen was terminated.

Polymyxin B exhibits rapid, concentration-dependent bacterial killing *in vitro* against a range of Gram-negative organisms including *K. pneumoniae* (Poudyal et al., 2008); and several reports suggest polymyxin B had a good clinical potential in bloodstream infection (Kvitko et al., 2011; Medeiros et al., 2019; Falcone et al., 2021). In the present study, microbiological eradication was achieved by the third day of treatment in all eight patients assessed and blood culture remained negative until they had completed the study or withdrew. Clinical improvement occurred in 6 of 8 (75%) of these patients, with all 4 who completed the full 14 days of treatment alive 28 days after therapy commenced. Although direct comparison with alternative therapies are lacking, clinical efficacy of this study was satisfied. Pooled mortality among patients with CRKP BSI receiving various treatment was 54.3% according to a meta-analysis (Xu et al., 2017). A retrospective study analyzing clinical efficacy of amikacin and its combinations for CRKP infection found that 30-day mortality was 34.5% (Rodrigues et al., 2021). Recent retrospective study about efficacy of ceftazidime-avibactam for CRKP BSI showed that 30-day mortality was around 25%, whether alone or combined with other antibiotics (Tumbarello et al., 2021). While acknowledging the small number of patients in our cohort, two factors may have contributed to this result. First, the SOFA score of our patient cohort, which is a risk factor for mortality generally and has been shown to be independently associated with mortality in patients with CRE BSI, was generally low (range, 1–10; median, 4; only one patient was receiving vascular-active drugs) (Falcone et al., 2021). Second, the rapid eradication of CRKP within 3 days of commencing therapy likely benefited the disease recovery process.

Our study is the first to specifically examine patients with BSI using the recommended dosage regimen of polymyxin B (2.5–3.0 mg/kg/d) (Tsuji et al., 2019). Previous studies utilizing PPK models to examine polymyxin B PK have included primarily critically ill patients with various medical conditions or patients with cystic fibrosis, and have employed a wide range of doses (0.45–3.38 mg/kg/day) (Sandri et al., 2013; Avedissian et al., 2018; Kubin et al., 2018; Manchandani et al., 2018; Miglis et al., 2018; Yu et al., 2021). Interestingly, it is noteworthy that the PK of polymyxin B in patients with BSI was very different from in healthy subjects (Liu et al., 2021). Although the clearance was similar in both groups (0.028 ± 0.007 L/kg/h in BSI patients and 0.026 ± 0.004 L/kg/h in healthy subjects), the volume of distribution and half-life in BSI patients were over twice that of healthy subjects (0.490 ± 0.142 L/kg vs. 0.204 ± 0.026 L/kg and 12.5 ± 3.11 vs. 5.55 ± 0.942 h, respectively). Increases in volume of distribution and half-life in critically ill patients are not surprising and was reported in other antibiotics previously (Bergen et al., 2011; Pea, 2013; Blot et al., 2014). However, when built PPK model from BSI patients, a full PPK model with covariates could not be built possibly due to a small number of patients. Considering plenty of studies built PPK models by combining data from all available trials (Lu et al., 2019; Gaudy et al., 2020; Naik et al., 2021), and population pharmacokinetic guidance by FDA also encourages combining data from early- and late-stages of trials to build models using the non-linear mixed-effects modeling approach (https://www.fda.gov/media/128793/download).We therefore included all plasma concentrations acquired from healthy subjects and BSI patients in the PPK model. Not surprisingly, BSI was included as a category covariate on the volume of the central compartment (V1), whereas CLcr before and during treatment was not included as a covariate in the model. On this latter point, polymyxin B is known to be predominantly non-renally cleared with ≤4% of the dose excreted unchanged in the urine (Zavascki et al., 2008). Although body weight has been included in several reported PPK models (Sandri et al., 2013; Xie et al., 2020), it was not incorporated as a covariate in our model most likely due to the relatively narrow range of body weight of enrolled subjects (range, 55–65 kg; median 60 kg). Although age is commonly considered as an underlying covariate of weight or progressive decline in the functional reserve of multiple organs and systems (ElDesoky, 2007; Klotz, 2009; Shi and Klotz, 2011), it is incorporated as a covariate that influences polymyxin B distribution (included on V2).

A good understanding of the PK of polymyxin B is essential for optimizing its clinical use. A previous population PK study examining the use of polymyxin B (0.45–3.38 mg/kg/day) in 24 critically ill patients reported an AUC_ss,24h_ of 66.9 ± 21.6 mg h/L (range, 16.4–117 mg h/L) and C_ss,avg_ of 2.79 ± 0.90 mg/L; no loading dose was administered (Sandri et al., 2013). In our present study utilizing a 2.5 mg/kg loading dose and 1.25 mg/kg maintenance dose of polymyxin B for BSI patients, non-compartmental analysis showed that the AUC_ss,24h_ (calculated as 2× AUC_ss,12h_) and C_ss,avg_ were 79.6 ± 25.0 mg h/L and 3.35 ± 1.06 mg/L, respectively, somewhat higher than the values reported by Sandri *et al.* and well within the recommended targets for AUC_ss,24h_ and C_ss,avg_ of 50–100 mgh/L and 2–4 mg/L, respectively (Tsuji et al., 2019).

Similar to colistin, *f*AUC/MIC has been shown to be the most predictive PK/PD index for polymyxin B (Lin et al., 2017; Landersdorfer et al., 2018). In preclinical studies, the *f*AUC/MIC required for various magnitudes of bacterial killing varied among bacterial strains and infection sites. In a mouse thigh infection model undertaken with three strains of *K. pneumoniae*, target values of *f*AUC/MIC for polymyxin B of 3.7–28.0 were required for 1 log_10_ kill, while 2 log_10_ kill could not be achieved with even the highest tolerated dose (Landersdorfer et al., 2018). Against *P. aeruginosa* and *A. baumannii*, an *f*AUC/MIC target for colistin of ∼17 has been established for 2 log_10_ kill in a mouse thigh infection model (Dudhani et al., 2010a; 2010b). A strength of our study is that our PK/PD results were obtained from the infection site. In our patient cohort with CRKP BSI, the AUC_ss,24h_/MIC was ≥54.4 (*f*AUC_ss,24h_/MIC of 22.8) in the eight patients in whom clinical efficacy was assessed, with microbiological eradication from the blood achieved in all patients within 3 days of commencing therapy.

Monte Carlo simulations showed that *f*AUC_ss,24h_/MIC targets of 13.5 for *K. pneumoniae* and 17.6 and 17.4 for *P. aeruginosa* and *A. baumannii*, respectively, were achieved in >90% of patients for pathogens with MIC ≤1 mg/L with our maintenance dosing regimen of 2.5 mg/kg/day (Figure 4). However, the likelihood of achieving these targets with this dosage regimen for organisms with an MIC of 2 mg/L was poor and remained <90% even with the maximum recommended daily dose of 3 mg/kg (except for *K. pneumoniae*). These results are very similar to those reported by Sandri et al. (2013) for critically ill patients generally. But these results should be interpreted with caution because the PK/PD target is based on small numbers of animal studies. For pathogens with MIC ≥2 mg/L it would seem prudent to use polymyxin B in combination with other antibiotics such as a carbapenem, tigecycline and fosfomycin to maintain efficacy and reduce toxicity (Zhang et al., 2017). Importantly, despite achieving microbiological eradication from the bloodstream in the eight assessed patients, intra-abdominal infection in two patients with low *f*AUC_ss,24h_/MICs (46.4 and 28.9) did not resolve and they subsequently died on days 11 and 45. Therefore, it is critical to determine the specific *f*AUC/MIC targets of polymyxins for different infection sites after intravenous administration.

There are some limitations in the present study. The relatively small number of BSI patients is limited for the clinical and microbiological efficacy evaluation. More patients are needed to enrolled in the study to validate the efficacy of the dosing regimens, as well as to collect more PK samples for the PPK model and PK/PD analysis.

## Conclusion

This is the first study to examine the clinical PK/PD of polymyxin B in BSI patients infected with CRKP. Polymyxin B administered at a recommended dose of 1.25 mg/kg 12-hourly achieved an AUC_ss,24h_/MIC of ≥54.4 and was sufficient to achieve microbiological eradication from the blood of all patients. Monte Carlo simulations indicated that the recommended dose of polymyxin B would be suitable for bloodstream infections caused by pathogens with an MIC ≤1 mg/L. These results will assist to establish polymyxin B breakpoints and to optimize its therapy in difficult-to-treat patients.

## Data Availability

The original contributions presented in the study are included in the article/Supplementary Material, further inquiries can be directed to the corresponding authors.
