# Supplementary material for: Pharmacokinetics/pharmacodynamics of polymyxin B in patients with bloodstream infection caused by carbapenem-resistant Klebsiella pneumoniae
Source: Front Pharmacol. 2022 Dec 16;13:975066. doi: 10.3389/fphar.2022.975066 (PMC9800617; doi:10.3389/fphar.2022.975066)
Supplement: Supplementary file 1 [file DataSheet1.PDF]

Table S1. Inclusion/exclusion criteria for intravenous polymyxin B in Chinese patients with carbapenem-resistant *K. pneumoniae* (CRKP) bloodstream infection.

**Inclusion criteria**
1. Patient with a clinical diagnosis of bloodstream infection and blood culture revealing carbapenem-resistant *K. pneumoniae* (CRKP);
2. ≥18 years old;
3. A negative pregnancy test (female patients), and consent to use effective contraceptive measures throughout the study (female and male patients);
4. Endogenous creatinine clearance in the range of 60-120 mL/min;
5. Voluntary enrolment in the study and signing informed consent.
**Exclusion criteria**
1. Hematology patient;
2. Administered systemic antibiotics for >48 hours with symptoms of infection alleviated;
3. Glasgow score ≤8 or life expectancy <less than 1 month;
4. History of allergy to polymyxins or meropenem;
5. Participation in another clinical trial in the past 3 months;
6. Unable to comply with the study protocol;
7. Potential safety issues will occur if following the protocol’s treatment or procedures;
8. History of epilepsy or myasthenia gravis;
9. Inability to collect PK blood samples;
10. Co-administered or expected to be co-administered nephrotoxic drugs during the study.

Table S2. Definition of population groups

**Population group**	**Definition**
Intention to treat (ITT)	All enrolled patients who signed the informed consent.
Modified ITT (mITT)	Enrolled patients who finished loading dose and steady state blood collections for polymyxin B pharmacokinetics according to the protocol.
Per-Protocol population (PP)	Enrolled patients who completed 14-days treatment with polymyxin B per-protocol and finished all the blood collections for polymyxin B pharmacokinetics and clinical evaluations including safety and efficacy according to the protocol.
Safety analysis set (SS)	Enrolled patients who received at least 1 dose of polymyxin B.

Table S3. Clinical outcome criteria

**Clinical outcome**	**Definition**
Clinical cure	All symptoms and signs of bloodstream infection when the patient enrolled have disappeared or returned to normal and do not require continued antibacterial treatment.
Clinical improvement	All or some symptoms and signs of bloodstream infection when the patient enrolled have improved but have not returned to normal and require continued antibacterial treatment.
Clinical failure	Symptoms and signs of bloodstream infection when the patient enrolled have not improved or have worsened and required continued antibacterial treatment.

Table S4. Drugs co-administered with polymyxin B in enrolled patients

Patient No.	Co-administered drug
1	Ademetionine, magnesium isoglycyrrhizinate, flurbiprofen, ambroxol, alprostadil, acetylcysteine, somatostatin, furosemide, albumin, magnesium sulfate, linezolid, micafungin, parecoxib
2	Paracetamol, aspirin, metoprolol, atorvastatin, cloprdogrel, insulin aspart, insulin glargine, methionine, indomethacin, rivaroxaban
3	Albumin, furosemide, somatostatin, flurbiprofen, esomeprozole, ademetionine, parecoxib, caspofungin
4	Ademetionine, pantoprazole, doxofylline, ambroxol
5	Esomeprazole, ursodesoxycholic acid, ademetionine, tamsulosin, Xueshuantong, paspertin flavoxate, Relinqing, paracetamol
6	Omeprazole, metoclopramide, low molecular heparin, celecoxib
7	Pantoprazole, amlodipine, haloperidol, quetiapine, olanzapine, citicoline, polysaccharide-iron complex injection
8	Linezolid, parecoxib, octreotidum
9	Pantoprazole, somatostatin, ambroxol, butorphanol, dexmedetomidine, ademetionine, albumin, diammonium glycyrrhizinate, milrinone, caspofungin, magnesium sulfate, alprostadil

Table S5. Renal function changes across the study period. Data are presented as estimated glomerular filtration rate (using CKD-EPI function, mL/min)/serum creatinine concentration (μmol/L).

No.	D1	D2	D3	D4	D5	D6	D7	D8	D9	D10	D11	D15	D16
1	107.9/52			99.1/64				47.8/135					
2	93.2/73			51.1/127		44.3/143							
3	99.2/52		94.8/58	100/51				97.6/54	95.5/57			88.3/69	
5	98.9/61			39.3/156		48.5/131							
6	92.3/64			87/74			87.5/73				88/72	91.2/66	
7	112.4/34			87/61			68.6/75				80.1/66		87/61
8	115.8/33			86.9/65									
9	70.8/89.4				76/84.3			85.5/70.8					

Note: Patient 4 withdrew from the study after the first dose and was excluded from the table. Patients 1, 2, 5, 7, and 8 experienced an increase in serum creatinine within 7 days (graded "I" according to the RIFLE criteria), with patients 1, 2, 5 and 8 withdrawing from the study on days 8, 6, 6 and 5, respectively.

Table S6. Pharmacokinetic parameters by two-compartmental analysis

**Parameter**	**Unit**	**Mean value ± SD**
K_{10}	1/h	0.165 ± 0.065
K_{12}	1/h	0.702 ± 0.395
K_{21}	1/h	0.214 ± 0.137
V_1	L/kg	0.163 ± 0.036
V_2	L/kg	0.565 ± 0.243
CL	L/h/kg	0.026 ± 0.008

Note: K_{10}, elimination rate constant; K_{12}, rate constant for distribution of polymyxin B from the central to peripheral compartment; K_{21}, rate constant for distribution of polymyxin B from the peripheral to central compartment; V_1, central volume of distribution; V_2, peripheral volume of distribution; CL, clearance from the central compartment.

Table S7. Progression of population pharmacokinetic model building

Run No	Database population	Structure and covariance model	OFV	AIC	BIC	Note
1	Patients	1CP	50	62	80	Successful
2	Patients	2CP	-288	-274	-253	Successful
3	Patients	2CP+Covariates	NA	NA	NA	Fail to get a stable model with a reasonable conditional number (>1000)
4	Patients+ healthy	1CP(Fail)	1160	1170	1189	Figures of DV vs PRED and DV vs IPRED were not in a diagonal relationship
5	Patients+ healthy	2CP	-988	-972	-941	Successful
6	Patients+ healthy	3CP	-1049	-1029	-990	Fail to get a conditional number
7	Patients+ healthy	2CP+DIS	-1034	-1031	-975	ΔOFV decreased by -46 compared with run 5
8	Patients+ healthy	2CP+DIS+ AGE	-1088	-1068	-1029	ΔOFV decreased by -54 compared with run 5

Note: CP: compartmental model; OFV: objective function valuel AIC: Akaike Information Criterion; BIC: Bayesian Information Criterion

Table S8 Probability of target attainment for different dosage regimens of polymyxin B against common carbapenem-resistant organisms according to PK/PD simulations.

Organism	Dose (mg/kg)	Dosing frequency	MIC (mg/L)					
			0.25	0.5	1	2	4	8
Klebsiella pneumoniae	2	Loading dose	100.0	100.0	99.6	40.5	0	0
	2.5		100.0	100.0	100.0	71.0	2.0	0
	0.5	Maintenance dose, q12h	100.0	99.7	40.5	0.2	0	0
	0.75		100.0	100.0	90.6	11.0	0	0
	1.0		100.0	100.0	99.6	40.7	0.1	0
	1.25		100.0	100.0	100.0	71.3	2.1	0
	1.5		100.0	100.0	100	91.1	11.0	0
Pseudomonas aeruginosa	2	Loading dose	100.0	100.0	92.7	12.0	0	0
	2.5		100.0	100.0	99.5	35.0	0.1	0
	0.5	Maintenance dose, q12h	100.0	92.8	12.5	0	0	0
	0.75		100.0	100.0	60.0	0.9	0	0
	1.0		100.0	100.0	92.7	12.9	0	0
	1.25		100.0	100.0	99.3	36.0	0	0
	1.5		100.0	100.0	100.0	60.0	0.8	0
Acinetobacter baumannii	2	Loading dose	100.0	100.0	93.3	12.7	0	0
	2.5		100.0	100.0	99.6	36.1	0.1	0
	0.5	Maintenance dose, q12h	100.0	93.5	13.3	0	0	0
	0.75		100.0	100.0	61.6	1.0	0	0
	1.0		100.0	100.0	93.3	13.9	0	0
	1.25		100.0	100.0	99.3	37.4	0	0
	1.5		100.0	100.0	100.0	61.6	0.9	0

Note: The *f*AUC/MIC targets were 13.5 for a 1-log10 reduction against *K. pneumoninae* and 17.6 and 17.4 for a 2-log10 reduction against *Pseudomonas aeruginosa* and *Acinetobacter baumannii*, respectively. The unbound fraction in plasma was 0.42.

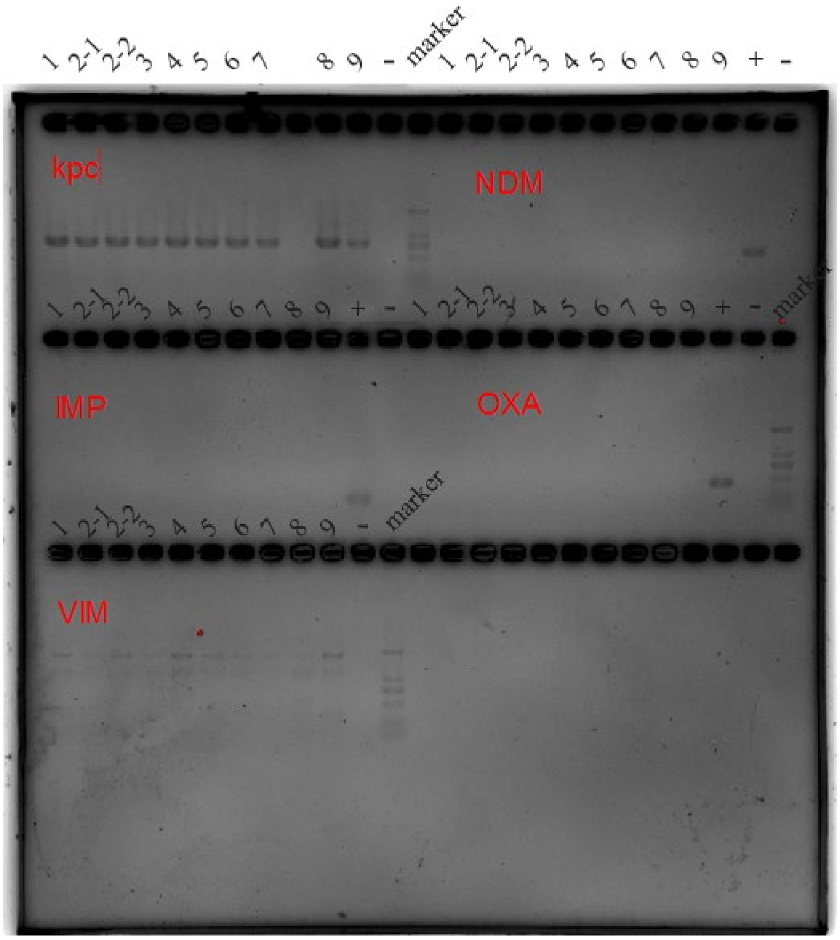

Fig S1. PCR results of antibiotic resistance genes

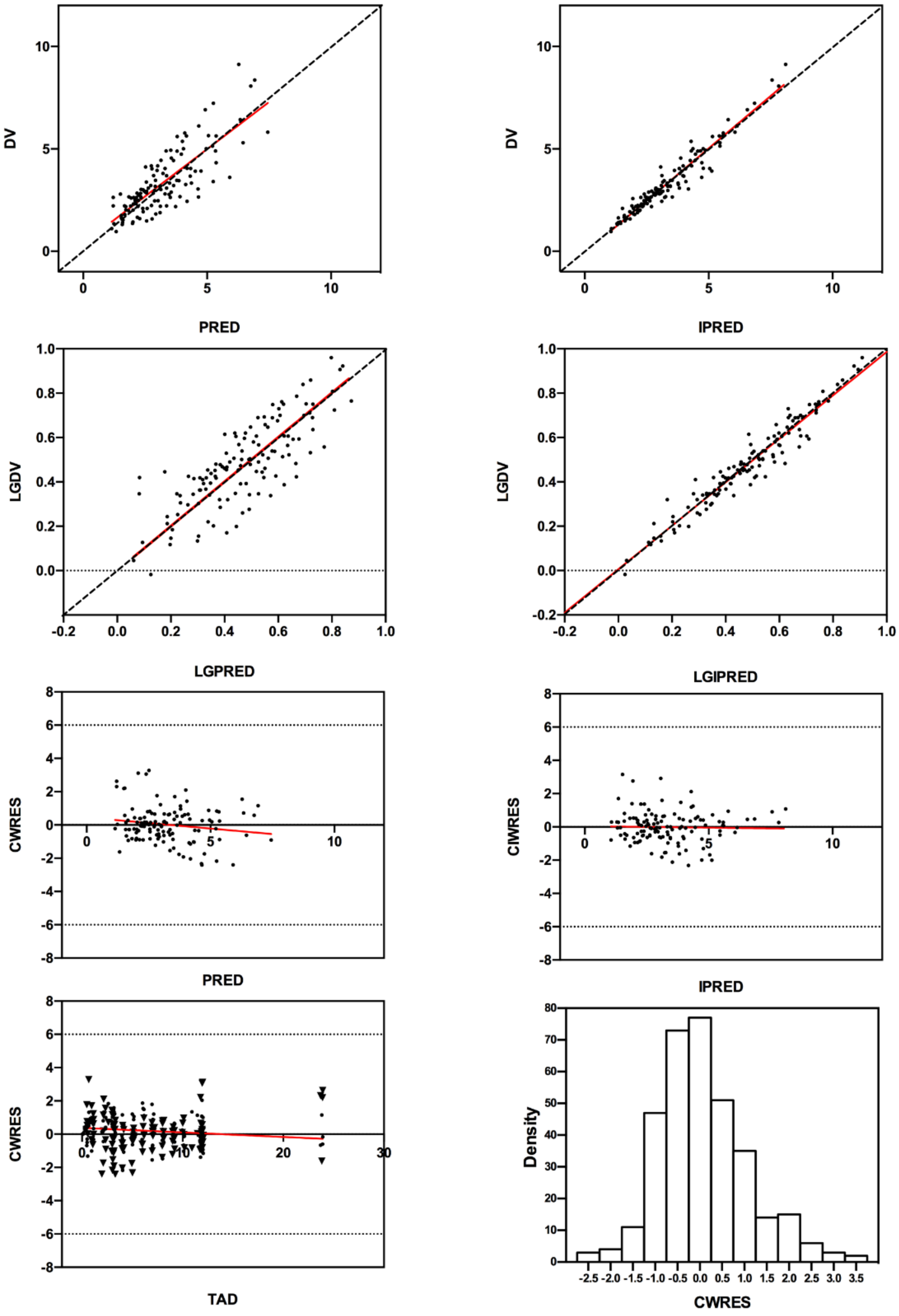

Fig. S2 Goodness-of-fit plots for the final population PK model for patients.

Predicted concentrations are in micrograms per liter and time is in hours. DV, Observed polymyxin B concentration; PRED, population predictions; IPRED, individual predictions; LGDV, Log transformed DV; LGIPRED, Log transformed IPRED; LGPRED, Log transformed PRED; CWRES, Conditional weighted residuals; CIWRES, Conditional individual weighted residuals; TAD time after dose.

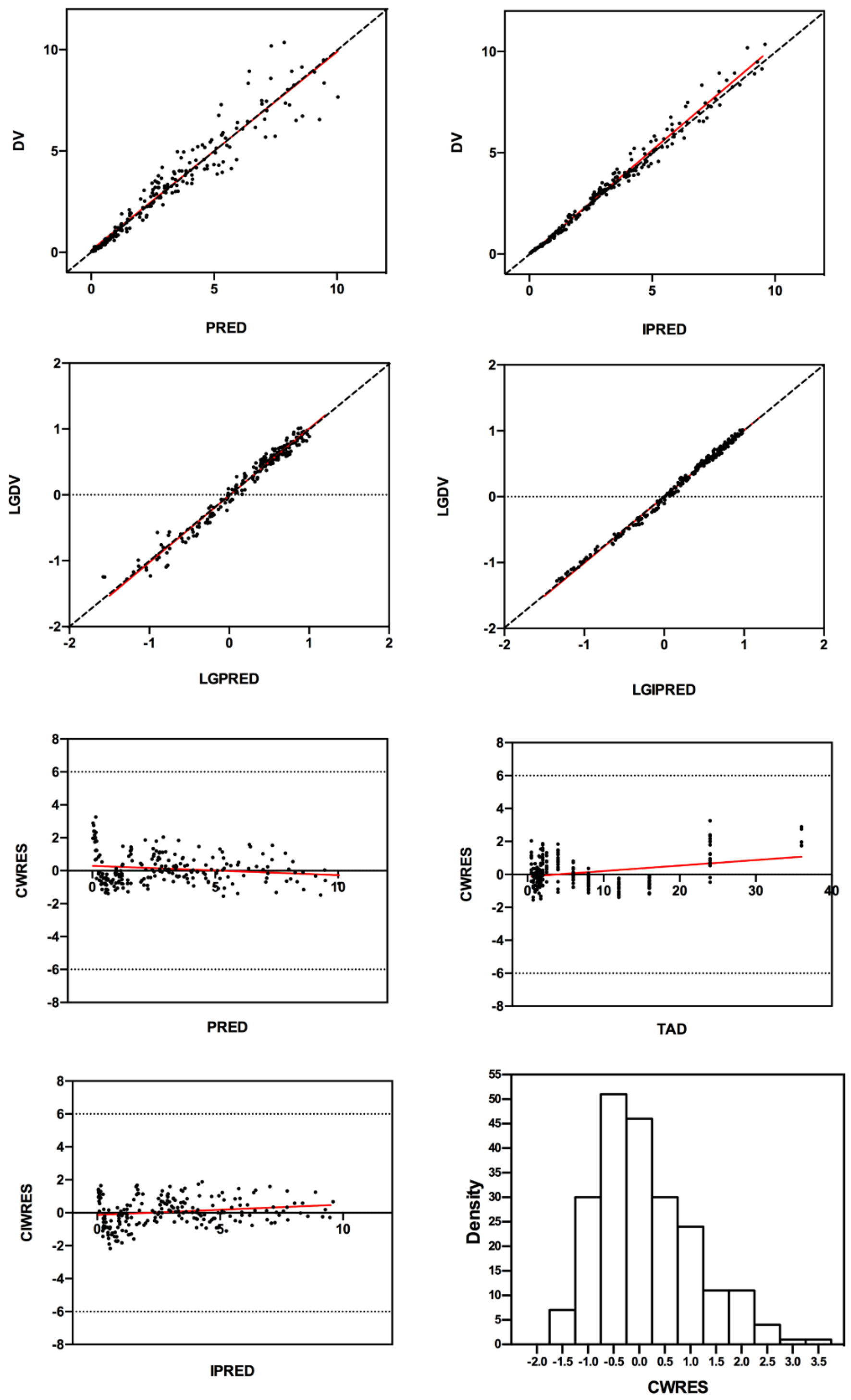

Fig. S3 Goodness-of-fit plots for the final population PK model for healthy subjects.

Predicted concentrations are in micrograms per liter and time is in hours. DV, Observed polymyxin B concentration; PRED, population predictions; IPRED, individual predictions; LGDV, Log transformed DV; LGIPRED, Log transformed IPRED; LGPRED, Log transformed PRED; CWRES, Conditional weighted residuals; CIWRES, Conditional individual weighted residuals; TAD time after dose.
